# Influence of biochar, mycorrhizal inoculation, and fertilizer rate on growth and flowering of *Pelargonium* (*Pelargonium zonale* L.) plants

**DOI:** 10.3389/fpls.2015.00429

**Published:** 2015-06-16

**Authors:** Giulia Conversa, Anna Bonasia, Corrado Lazzizera, Antonio Elia

**Affiliations:** Department of the Science of Agriculture, Food and Environment, University of FoggiaFoggia, Italy

**Keywords:** mycorrhizal root colonization, bulk density, porosity, dry weight, mineral content, electrolyte leakage, relative water content, chlorophyll

## Abstract

Peat is the most common substrate used in nurseries despite being a very expensive and a non-renewable material. Peat replacement with biochar could be a sound environmental practice, as it is produced from waste biomass, but evaluation of biochar as a potting substrate is needed. Ratios of peat:biochar of 100:0, 70:30, 30:70 (BC_0_, BC_30_, and BC_70_, respectively), two fertilizer rates (FERT_1_, FERT_2_), and arbuscular mycorrhizal fungi (AMF) inoculation were tested on potted *Pelargonium* plants. Plant growth, flowering, bio-physiological and nutritional responses, and root mycorrhization were evaluated. The BC_30_ mixture did not affect plant growth compared with pure peat. However, BC_30_ in combination with FERT_2_ treatment was more effective in enhancing nitrogen (N) and chlorophyll (CHL) leaf concentrations, and leaf and flower numbers. The BC_70_ mixture depressed plant growth, flowering traits, and root mycorrhization. Leaf N concentration was below the sufficiency range reported for *Pelargonium* growth. Leaf concentration of phosphorous (P) was adequate in pure peat and in BC_30_ but it dropped close to sub-optimal values in BC_70_. The pH value of the mixtures lowered P availability, though in BC_30_ the mycorrhizal activity could have allowed adequate P plant uptake. In BC_70_ plants, the deficiency of both N and P might be a reason for the observed growth reduction. The inoculation of the substrate with selected AMF improved plant growth (higher dry biomass, greater floral clusters, larger and more abundant leaves) and quality resulting in unstressed (lower electrolyte leakage and higher relative water content values) and greener leaves (low L^∗^ and C^∗^, high CHL content) and in more intensely colored flowers. We conclude that biochar can be applied in nursery/potted plant production provided that the proportion in the peat mixture does not exceed 30%. Furthermore, AMF inoculation contributed to achieving the best plant performance in 30% biochar amended medium.

## Introduction

Biochar (BC) is produced via hydrothermal carbonization from a wide range of biomass sources including agricultural wastes, green-waste, and animal manures (biomass feedstock). Many studies have examined the addition of biochars to soil systems, with both positive and negative results (Spokas et al., 2012). The positive effects of biochar application to agricultural soils are linked to changes in soil physical and chemical parameters leading to increased water-holding capacity, reduction of bulk density (BD), and addition of cation exchange sites (Laird, 2008). Other studies on biochar amended soil have reported positive interactions between biochar and application of N and P fertilizer on plant growth (Van Zwieten et al., 2010), which were attributed to reduced leaching and hence more efficient use of applied nutrients (Sohi et al., 2010; Laird et al., 2011). Furthermore, by serving as a source of reduced carbon compounds and by increasing the availability of micronutrients, biochar may be beneficial to microbial populations such as arbuscular mycorrhizal fungi (AMF; Warnock et al., 2007; Solaiman et al., 2010; Lehmann et al., 2011). However, above a certain threshold of application in the soil, biochar may decrease AMF root colonization (Warnock et al., 2010; Hale et al., 2013; LeCroy et al., 2013; Mukherjee and Zimmerman, 2013).

Peat is the most common substrate used in horticultural nurseries because of its good chemical and physical properties such as low pH, high cation exchange capacity, low inherent fertility, proper balance of aeration and water-holding porosity, and sufficient rigidity to support the plant (Bilderback et al., 2005), although it is reported to be usually poor in AMF (Navarro et al., 2011, 2012). Peat is a very expensive and a non-renewable material, and the research for finding low-cost and environmentally friendly alternative products is encouraged (Ostos et al., 2008; Moral et al., 2009; Farrell and Jones, 2010; Jayasinghe et al., 2010b; Vaughn et al., 2011).

The potential benefits of biochar for plant growth may be leveraged in the production of potted plants but only a few studies have used biochars as a replacement for peat in potting substrates. Plant responses to the addition of biochar to substrates can be similar to those found with standard substrates containing peat (Dumroese et al., 2011; Altland and Locke, 2012; Tian et al., 2012; Vaughn et al., 2013) with the benefits of creating a beneficial environment for microbes (Graber et al., 2010). The negative effects of biochar soil amendment are linked to its high alkalinity (Ca, Mg, K, and Si hydroxides and carbonates present in the biomass feedstock) and to high concentrations of leachable polyaromatic hydrocarbons, which are potentially toxic (Rogovska et al., 2012). The concentration of these polyaromatic hydrocarbons is positively related to charring temperature therefore the use of biochar from gasification (the high temperature -600/1400°C- partial combustion of biomass) is potentially more problematic than biochar produced via pyrolysis methods (Laird et al., 2011).

Gasification of industrial wastes, municipal solid wastes, and even biomass is already widely used because it involves an exothermic release of heat, which can be captured (Peterson and Jackson, 2014). Potting substrate experiments based on biochar (up to 15% in peat mixtures) produced by gasification have been successfully carried out on tomato and marigold plant cultivation (Vaughn et al., 2013).

The main objectives of this research were to evaluate the effect of biochar on plant nutrient availability and on native and/or selected AMF activity in the potted plant production process. To achieve these objectives we grew potted *Pelargonium* plants in substrates with three peat:biochar ratios, with two rates of fertilizer, inoculated or not with selected AMF and assessed plant growth, flowering traits, total chlorophyll (CHL) concentration, electrolyte leakage (EL), relative water content (RWC), nutritional responses, and quality.

## Materials and Methods

### Transplants, Peat, Biochar, Arbuscular Mycorrhizal Fungi Inoculum, and Fertilizer

*Pelargonium* (*Pelargonium zonale* L., cv Pinnacle Dark Red) rooted stem-cuttings were obtained from Lazzeri Agricultural Group (Merano -BZ, Italy). Plants were about 13 cm tall and had nine leaves (989 cm^2^ area), with a fresh leaf weight of about 49 g, and a fresh root weight of about 9 g. Peat (Type 3 special modified-Brill, Georgsdorf, Germany) was a mixture (1:1 v:v) of light and dark moss peat with 500 g m^-3^ of fertilizer added (160, 160, 180 mg L^-1^of N, P_2_O_5_, and K_2_O, respectively). The biochar [Agrindustria S.N.C., Frazione Roata Rossi, Cuneo (CN), Italy] feedstock consisted of ground (2–7 cm particle size) and dried (humidity ≤ 10%) whole tree *Abies alba* (Mill.). Feedstock underwent pyro-gasification in the Romana Maceri 200 kW plant (RM Impianti SrL, Arezzo) at a reactor temperature of 1,000–1,100°C which produced small amounts (5%) of biochar.

At the beginning of the trial the characterization of biochar was performed (six replicates). Biochar pH and EC were both measured in water extracts (sample:distilled water ratio of 1:5, w/v) using a glass electrode and an EC meter (HI 991301, Hanna Instruments; Jayasinghe et al., 2010a). The cation exchange capacity was estimated by the barium chloride-triethanolamine method (Official method no. XIII. 2, Ordinary Suppl. Italian G.U. No. 248 of 21/10/1999) which was buffered at pH 8.2. Biochar samples were oven-dried at 70°C for 24 h, ground to pass through a <1 mm sieve before analysis for nutrients. The C and N concentrations were determined using a CHN Elemental Analyzer (Thermo Flash EA 1112). For the determination of total concentrations of P, K, Ca, Na, Fe, Mg, and Mn, the samples were ashed in a muﬄe furnace at 500°C for 2 h then the ash was dissolved in 200 mL 10% HCl. The solutions obtained were filtered (0.45 μm PTFE) and diluted. Concentrations of P, K, Ca, Na, Fe, Mg, and Mn were determined using an ICP optical spectrometer (Varian Inc., Vista MPX). The AMF inoculum was a commercial product (Aegis SymMicrogránulo– Atens Agrotecnologias Naturales S.L., Tarragona, Spain) containing isolates of *Glomus intraradices* (Schenck and Smith) and *Glomus mosseae* [(Nicol. and Gerd.) Gerdemann and Trappe]. The inoculum consisted of mixed rhizosphere samples from plant cultures containing 25 spores per gram of each fungus, hyphae, and heavily infected root fragments with many internal spores. The inoculum was multiplied on leek (*Allium porrum* L.) under greenhouse conditions in Terragreen^®^ substrate. This calcined clay (average particle size 5 mm) [Oil-Dri. US special Ty/IIIR (Oil-Dri. Company, Chicago, IL, USA)] is an attapulgite from Georgia (USA) used as a substrate for propagation of AMF (Plenchette et al., 1996).

### Plant Growing Conditions, Treatments, and Experimental Design

The experiment was carried out in a growth chamber (A.T, Applied Technosystem, Bari, Italy) at the Department of the Science of Agriculture, Food and Environmental (SAFE) of the University of Foggia from November 2012 to January 2013. The growth chamber temperature was 23/18°C day/night, with a 10 h photoperiod, 70–80% relative humidity and 35,000 lx light intensity. Irradiance illumination was supplied by Sylvania (Canada), cool white high intensity fluorescent lamps, supplemented with 60 W incandescent lamps.

The trial included the following experimental treatments: (i) substrate mixtures, peat:biochar 100:0, peat:biochar 70:30 (v:v), peat:biochar 30:70 (v:v) indicated as BC_0_, BC_30_, and BC_70_, respectively; (ii) fertilizer (FERT) rates indicated as FERT_1_ and FERT_2_; (iii) the media AMF inoculation (MICO+) and the not inoculated substrate (MICO_0_).

Before preparation of the mixtures, biochar was passed through a < 2 cm sieve. The slow release fertilizer Nitrophoska^®^ Gold^®^ (15-9-15; COMPO Agro Specialities Srl. Cesano Maderno, MB, Italy) was added at a rate of 140 and 210 mg L^-1^ of medium during media preparation. AMF inoculum was applied at the FERT_1_ and FERT_2_ mixtures using 12 kg m^-3^ of media, as recommended by the producer. The substrates were not inoculated for MICO_0_ treatment.

The experimental design was a fully factorial randomized block design with four replicates. Each experimental unit (plot) was represented by 12 pots each containing one *Pelargonium* plant.

On October eighth 2012 the rooted cuttings were transplanted into 0.70 L conical-trunk shaped pots with eight holes in the bottom (ARCA S.P.A, Osio Sotto, BG, Italy).

The pots were bottom watered with tap water (pH 7.6; EC 0.70 mS cm^-1^). Irrigation was managed by tensiometers (2725 ARL Jet Fill Tensiometer, Soil moisture Equipment Corp., Santa Barbara, CA, USA) placed in four pots for BC_0_, BC_30_, and BC_70_ treatments. In each substrate, treatment irrigation started when the mean water tension reached 20 cbars.

### Sampling and Plant Analysis

Twenty-eight days after the transplant (DAT) and at the end of the trial (74 DAT) six plants per experimental plot unit were randomly sampled to determine (i) the bio-morphological parameters: fresh and dry weight (DW) of leaves, stems, floral clusters, and roots, the length of the main stem intended as plant height, the area and the number of the leaves, the number of branches, floral clusters, and flowers, and (only at 74 DAT) the leaf and flower color; (ii) the bio-physiological and nutritional parameters: the EL, the RWC, leaf CHL concentration, and the N, P, and K leaf concentration (only at 74 DAT). In order to determine the DW, plant fresh material was dried in a thermo-ventilated oven at 70°C until reaching a constant mass and successively finely ground through a mill (IKA, Labortechnik, Staufen, Germany) with a 1.0 mm sieve. Leaf area was determined using a leaf area meter (Li-3100, Licor, Lincoln, NE, USA).

The nitrogen, phosphorus, and potassium concentrations were analyzed on dried leaf material. Nitrogen content was determined through dry combustion (Dumas method) using a CHN Elemental Analyzer (Thermo Flash EA 1112). Phosphorus was determined through the molybdenum-blue method using a spectrophotometer (Shimadzu UV-1800, Shimadzu Scientific Instruments, Columbia, MD, USA) and the procedure described in Conversa et al. (2013). Potassium was determined by ion chromatography (Dionex ICS 3000; Dionex, Sunnivale, CA, USA) according to the method reported by Bonasia et al. (2010).

The total CHL, the EL, the RWC determination procedures details are reported in (Bonasia et al., 2013). Briefly, CHL was extracted from six leaf blade disks (taken from different plants), previously frozen and stored in the dark at -18°C until analyzed, by homogenizing in acetone 80%. The absorbance of the extract was measured at 647 and 664 nm, using a spectrophotometer (Shimadzu UV-1800) and expressed on a leaf fresh weight basis. The EL was determined as EL (%) = (EC_1_/EC_2_)*100 where, EC_1_ and EC_2_ were the electrical conductivity of, respectively, an un-boiled and boiled fresh leaf material solution. The RWC was determined on leaf disks as RWC = (FW-DW)/(TW-DW) where FW, blade fresh weight; TW, fully turgid blade weight; DW, blade dry weight. Color indices were measured on leaf and petal blade (two readings each per plant for six plants) using a portable tri-stimulus color-meter (Minolta Chroma Meter CR-200; Minolta Camera Co. Ltd., Osaka, Japan), using the CIE-L*a*b* scale 1976. The color intensity or saturation (C*) the hue angle (h°) were calculated by trigonometric functions (Bonasia et al., 2013).

For estimation of root mycorrhizal colonization, six root systems, collected from the plants used for aerial measurements, were taken at 28 and 74 DAT. Mycorrhizal colonization was assessed according to Conversa et al. (2013). Briefly, root segments (30) were randomly taken from each cleared and stained root system according to Phillips and Hayman (1970), and they were observed under ×100 magnification (Zeiss Primo Star Hal, Carl Zeiss S.P.A., Arese, MI, Italy). Mycorrhizal colonization was calculated by three parameters described by Trouvelot et al. (1986) and elaborated using *MycoCalc* software (http://www2.dijon.inra.fr/mychintec/Mycocalc-prg/download.html):

– F (%), frequency of mycorrhiza in the root system;
– M (%), intensity of mycorrhizal colonization in the root system;
– A (%), arbuscule abundance in the root system.

### Potting Mixture Characterization

At the beginning of the experiment, before adding fertilizer and/or AMF inoculum, the pH and EC of the media were measured as described above for biochar. Samples (six replicates) of 65°C oven-dried media were passed through a series of sieves, from 2 to 0.25 mm, to determine their particle-size distribution.

The ring knife method (Tian et al., 2012) was used to measure the BD, water-filled porosity, total porosity (TP), and air space (AS) of the mixtures. First, a ring knife with weight W_0_ and a volume of 400 cm^3^ was filled with air dried medium and weighed (W_1_). After the medium had been saturated with distilled water by soaking for 24 h, it was weighed again (W_2_). The ring knife with saturated medium was opened from one side (covered with gauze), placed on a holder with a leaky screen, and the water was allowed to drain from the medium for 3 h before the ring knife and medium were weighed again (W_3_). Finally, oven dried medium and the ring knife were kept at 65°C until they reached a constant weight and that weight (W_4_) was recorded. The following formulas were used to calculate medium characteristics: BD (g cm^-3^) = (W_4_-W_0_)/400; TP (%) = (W_2_-W_4_)/400 × 100; AS (%) = (W_2_-W_3_)/400 × 100; water-filled porosity (%) = TP-AS. At 74 DAT about 100 g of substrate were sampled from the bulk obtained from six pots. Samples were dried until constant weight in an oven at 110°C for determining the final water content.

### Statistical Analysis

All data were subjected to ANOVA using the general linear model (GLM) procedure (S.A.S v. 9.0 software – SAS Institute, Cary, NC, USA). A completely randomized design scheme was used for processing substrate composition data and a randomized block design for processing the data of plant characters. Differences between means were compared using the Least Significant Difference (LSD) test at *P* = 0.05. Before ANOVA analysis data were first tested for normal distribution and variance homogeneity with Kolmogorov–Smirnov test and the Levene test, respectively. When necessary, data were subjected to square root (for leaf, stem, and floral cluster number) or arcsin √x (for EL, RWC, F, M, and A) transformation before data analysis.

## Results

### Biochar Chemical Characteristics

The biochar used in the study had pH 10.7 (±0.05), EC 1.6 (±0.05) mS cm^-1^, cation exchange capacity 32.4 (±0.52) cmol(+) kg^-1^, carbon concentration of 794.2 (±11.39) g kg^-1^, nitrogen concentration of 2.9 (± 0.62) g kg^-1^, and C/N ratio 272. Other mineral elements concentration (mg kg^-1^) was: P 590 (±35.5), Ca 18,698 (±1,224), K 5,063 (±113), Mg 2,486 (±574), Na 173 (±10.31), Fe 172 (±29.08), and Mn 292 (±13.19). Ash content was 2.51 (±0.48) g kg^-1^.

### Effects of Biochar Amendment on Media Granulometric, Chemical, and Physical Characteristics

Irrespective of its proportion in the mixtures, biochar significantly lowered the fraction of 2.0–0.7/0.7–0.5 mm particles and increased 0.25–0.1 mm particles (**Table 1**). Biochar addition also increased >2 mm particles, this size class being the most represented in BC_70_ media.

**Table 1:** Weight of different particle-size classes of media measured before *Pelargonium* growing cycle.

Medium	Size fraction					
	>2.0 mm (g 100 g^-1^)	2.0–0.7 mm (g 100 g^-1^)	0.7–0.5 mm (g 100 g^-1^)	0.5–0.25 mm (g 100 g^-1^)	0.25–0.1 mm (g 100 g^-1^)	<0.1 mm (g 100 g^-1^)
BC_0_	19.6^c^	31.8^a^	28.8^a^	17.6^a^	2.3^b^	0.0^c^
BC_30_	30.1^b^	22.5^b^	9.1^b^	19.3^a^	13.0^a^	6.0^b^
BC_70_	42.8^a^	18.4^b^	6.0^b^	11.6^b^	12.9^a^	8.4^a^
Significance^1^	^∗∗^	^∗^	^∗∗∗^	^∗^	^∗∗^	^∗∗∗^

^1^^∗^, ^∗∗^, and ^∗∗∗^ significant at *P* ≤ 0.05, *P* ≤ 0.01 and *P* ≤ 0.001, respectively.

^a-b-c^Means (*n* = 6) in columns followed by the same letters are not significantly different according to the least significant difference (LSD) test (*P* = 0.05).

Biochar significantly affected the main chemical and physical characteristics of the mixtures (**Table 2**). The pH of BC_30_ and BC_70_ were almost one pH unit greater compared with the pure peat (BC_0_). Biochar increased EC (with the highest values being recorded in BC_70_), BD, AS and, when added at the highest proportion, it reduced TP and the water-filled porosity (WFC) of the substrate. The water content of potted media at the end of the experiment was the lowest in the substrate with the highest proportion of biochar (**Table 2**).

**Table 2:** Main characteristics of the containerized media measured at the beginning of trial.

Media	pH (1:5_H2O_)	EC (dS m^-1^)	Bulk density (g cm^-3^)	Total porosity (%)	Air space (%)	Water-filled porosity (%)	Final water content^4^ (g 100 g^-1^)
Ideal medium^1^			0.40 or less	>85	20–30	55–70	
BC_0_^2^	7.8^b^	0.49^c^	0.13^c^	84.1^ab^	12.9^b^	71.2^a^	76.7 a
BC_30_	8.6^a^	0.63^b^	0.14^b^	85.8^a^	18.1^a^	67.7^b^	73.3 b
BC_70_	8.7^a^	1.52^a^	0.16^a^	79.2^b^	21.4^a^	57.8^c^	67.4 c
Significance^3^	^∗^	^∗^	^∗∗∗^	^∗^	^∗∗^	^∗∗∗^	^∗∗∗^

^1^According to de Boodt and Verdonck (1972).

^2^BC_*0*_ = 100% peat, BC_*30*_ = peat:BC 70:30 (v:v); BC_*70*_ = peat:biochar 30:70 (v:v).

^3^^∗^, ^∗∗^, and ^∗∗∗^ significant at *P* ≤ 0.05, *P* ≤ 0.01 and *P* ≤ 0.001, respectively.

^4^Potted media water content at 74 days after the transplant (DAT).

^a-b-c^ Means (*n* = 6) in columns followed by the same letters are not significantly different according to the LSD test (*P* = 0.05).

### Effect of Biochar, Fertilizer Rate, and AMF Inoculation on Mycorrhizal Colonization

Root colonization by AMF was not detected at 28 DAT, but only at the end of the experiment, both in inoculated and non-inoculated media, indicating that indigenous AMF inoculum was already present in the peat (**Table 3**). AMF inoculation (MICO+) significantly increased mycorrhizal frequency (F) and intensity (M). The substrate with 70% biochar reduced mycorrhizal frequency (F; 5.7%) compared with BC_0_ and BC_30_ medium (14%, on average), while mycorrhizal colonization parameters were not affected by fertilizer rate (**Table 3**).

**Table 3:** Mycorriza colonization frequency (F) and intensity (M), and arbuscule abundance (A) of *Pelargonium* plants inoculated (MICO+) or not (MICO_0_) with arbuscular mycorrhizal fungi (AMF) and grown for 74 d in substrate amended with different rates of biochar (BC_0_, BC_30_, BC_70_), and fertilized with different rates of fertilizer (FERT_1_, FERT_2_).

Treatments	Mycorrhization parameter		
	F (%)	M (%)	A (%)
**Biochar rate (BC)^1^**
BC_0_	15.0^a^	0.48	0
BC_30_	13.0^a^	1.31	0
BC_70_	5.7^b^	1.12	0
**Fertilization rate (F)^2^**
FERT_1_	13.3	1.42	0
FERT_2_	9.7	0.51	0
**AMF inoculation (I)**
MICO+	14.6^a^	1.71^a^	0
MICO_0_	8.2^b^	0.14^b^	0
**Significance^3^**
Biochar rate	*	NS	NS
Fertilization rate	NS	NS	NS
AMF inoculation	*	^∗∗^	NS
BC*I	NS	NS	NS
BC*F	NS	NS	NS
I*F	NS	NS	NS
BC*I*F	NS	NS	NS

^1^BC_0_, BC_30_, and BC_70_: 100:0, 70:30, and 30:70 (v:v) peat:biochar substrate mixtures, respectively.

^2^FERT_1_ and FERT_2_: fertilized with 140 and 210 mg L^-1^ of Nitrophoska^®^ Gold^®^, respectively.

^3^NS, *, and ** not significant or significant at *P* ≤ 0.05 and *P* ≤ 0.01, respectively.

^a-b^Means in columns followed by the same letters are not significantly different according to LSD test (*P* = 0.05).

Means in columns without letters are not significantly different according to the LSD test (*P* = 0.05).

### Effect of Biochar and Fertilizer Rate on Plant Growth, Morphology, and Flowering Traits

**FIGURE 1:**
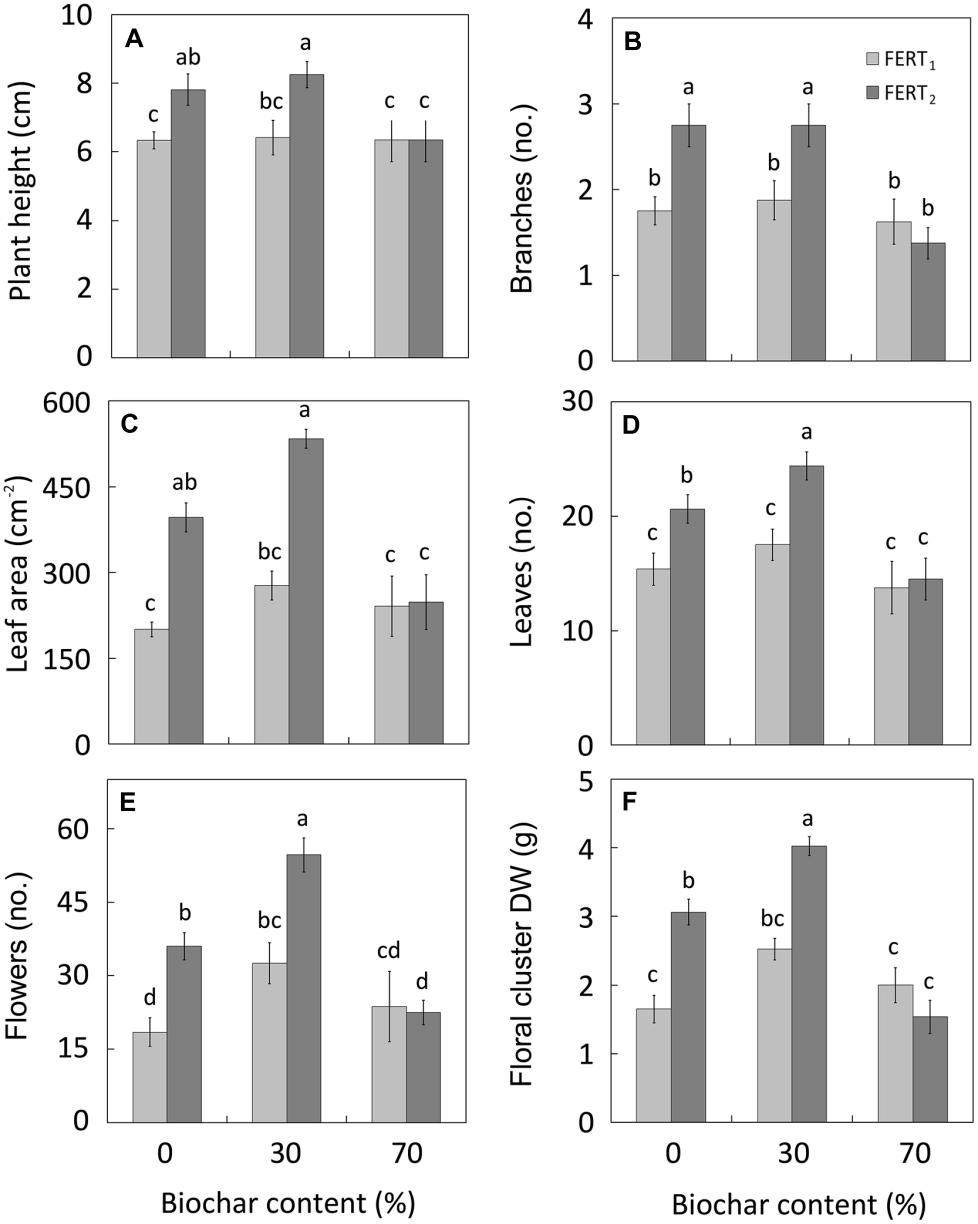
**Effect of peat:biochar ratio and fertilization rate on plant height **(A)**, branch number **(B)**, leaf area **(C)**, leaf **(D)** and flower number **(E)**, and floral cluster dry weight **(F)** of *Pelargonium* at the end of the experiment (74 DAT).** Bars indicate standard errors (*n* = 8). Treatments with different letters are significantly different according to the LSD test (*P* = 0.05). In **(B,D** and **E)**, histograms refer to the original data, while the used mean separation refers to the ANOVA performed on the arcsin 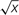 transformation of these data.

At both sampling dates, significant BC*FERT interactions emerged for many of the examined characteristics (**Table 4**). Irrespectively of biochar addition, the lower fertilizer rate had little effect on the final plant growth, while the higher fertilizer rate improved shoot growth of *Pelargonium* plants, except in the substrate with 70% biochar (**Figure 1**). Plants grown in BC_0_ and BC_30_ media and fertilized at the highest rate were the tallest (8 cm; **Figure 1A**), with the greatest number of branches (2.7 per plant; **Figure 1B**), and leaf area (534 cm^2^; **Figure 1C**). In particular, the BC_30_/FERT_2_ grown plants had the highest leaf (**Figure 1D**) and flower number (**Figure 1E**), and floral clusters DW (**Figure 1F**).

**Table 4:** Significance of *F* test for media, AMF inoculation, and fertilization rate treatments on container-grown *Pelargonium* vegetative structure and flowering traits after 28 and 74 DAT.

Treatments	Leaf area (cm^2^/plant)	Leaf (no./plant)	Total DW (g)	Leaf DW (g)	Stem DW (g)	Floral cluster DW (g)	Root DW (g)	Leaf area (cm^2^/plant)	Plant height (cm)^1^	Total DW (g)	Leaf DW (g)	Stem DW (g)	Floral cluster DW (g)	Root DW (g)	Leaf (no./plant)	Stem (no./plant)	Floral cluster (no./plant)	Flowers (no./plant)	Flowers (no./cluster)
	
	Sampling time (DAT)																		
	28							74											
Biochar rate (BC)	**	**	***	***	***	NS	*	*	NS	**	*	***	***	**	*	*	***	***	**
Fertilizer rate (F)	***	NS	NS	*	NS	NS	*	***	**	***	***	**	***	NS	***	**	*	***	*
AMF inoculation (I)	NS	NS	NS	NS	NS	NS	NS	**	NS	*	*	*	**	NS	*	NS	NS	**	*
BC*F	***	**	*	***	NS	NS	NS	***	*	**	*	NS	**	NS	*	*	NS	**	NS
BC*I	NS	NS	NS	NS	NS	NS	NS	NS	*	NS	NS	NS	NS	NS	NS	NS	NS	NS	NS
F*I	NS	NS	NS	NS	NS	NS	NS	NS	NS	NS	NS	NS	NS	NS	NS	NS	NS	NS	NS
BC*I*F	NS	NS	NS	NS	NS	NS	NS	NS	NS	NS	NS	NS	NS	NS	NS	NS	NS	NS	NS

^1^Length of the main stem corresponding to the plant height.

NS, *, **, and *** not significant or significant at *P* ≤ 0.05, *P* ≤ 0.01, and *P* ≤ 0.001, respectively.

**FIGURE 2:**
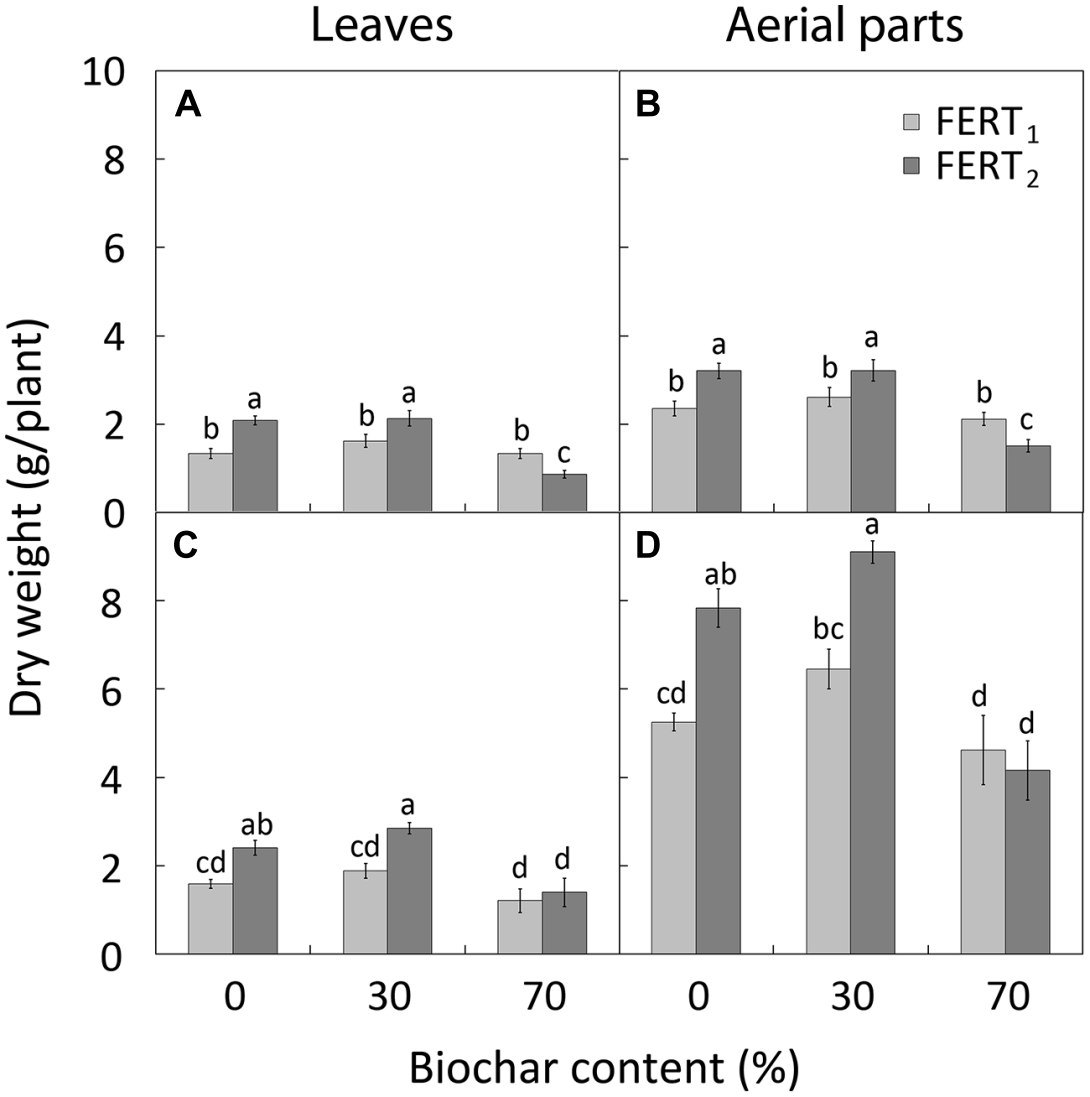
**Effect of peat:biochar ratio and fertilization rate on leaf and total DW of *Pelargonium* plants at mid-season (28 DAT; **A** and **B**, respectively) and at the end of the cycle (**C** and **D**, respectively).** Bars indicate SEs (*n* = 8). Treatments with different letters are significantly different according to the LSD test (*P* = 0.05).

Increasing the fertilizer rate increased the leaf and the total DW accumulation in 0 and 30% biochar added media at both 28 (**Figures 2A,B**) and 74 DAT (**Figures 2C,D**). At 28 DAT, BC_70_/FERT_2_ medium depressed leaf area and number (data not shown, treatment significance in **Table 4**) and the DW accumulation both in the leaves and in total aerial parts (**Figures 2A,B**). At both sample dates BC_70_ reduced stem DW (0.22 vs. 0.37 g/plant at 28 DAT and 0.6 vs. 1.2 g/plant at 74 DAT) and root DW (0.29 vs. 0.40 g/plant at 28 DAT and 0.6 vs. 1.1 g/plant at 74 DAT). BC_30_ plants produced the greatest number of floral clusters (5.0 vs. 3.7 clusters/plant) and the largest floral clusters (8.7 vs. 6.2 flowers/cluster) (data not shown, treatment significance in **Table 4**).

Increasing fertilizer rate reduced the initial root DW (0.32 vs. 0.41 g/plant), and improved the stem DW at the end of the experiment (1.1 vs. 0.8 g/plant), as well as the cluster formation (4.5 vs. 3.8 clusters per plant), and size (8.2 vs. 6.4 flowers per cluster; data not shown, treatment significance in **Table 4**).

### Effect of Biochar and Fertilizer Rate on Plant Bio-Physiological Response and Nutritional Status

**FIGURE 3:**
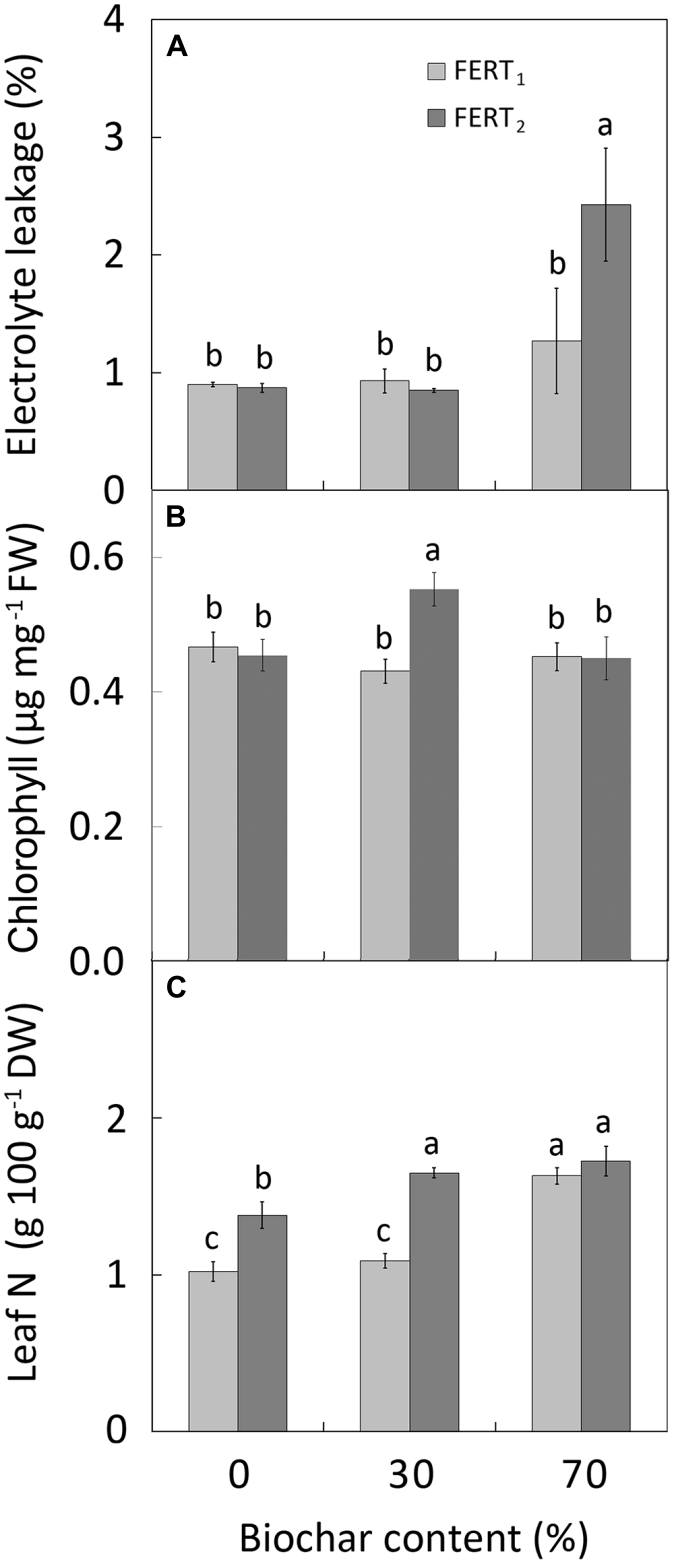
**Effect of peat:biochar ratio and fertilization rate on electrolyte leakage (at 28 DAT; **A**), chlorophyll content **(B)**, and N concentration **(C)** at the end of the experiment in *Pelargonium* plants.** Bars indicate standard errors (*n* = 8). Treatments with different letters are significantly different according to the LSD test (*P* = 0.05). In **(A)**, histogram has been done using the original data, while the used mean separation refers to the ANOVA performed on the arcsin 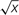 transformation of these data.

The EL of leaf tissues was affected by biochar and fertilizer treatments at 28 DAT, but not at 74 DAT (**Table 5**). In particular, at 28 DAT plants in the BC_70_/FERT_2_ treatment showed the greatest EL values (**Figure 3A**). Biochar amendment to the substrate only affected leaf RWC at 74 DAT, with the highest values in BC_70_ plants (**Table 5**). At 74 DAT, increasing fertilizer rate increased the total and the b CHL content (**Table 5**). However, the greatest CHL concentrations (0.39, 0.16, and 0.55 μg mg^-1^ for CHL a, b and total, respectively) were observed in leaves from BC_30_ plants (**Figure 3B**).

**Table 5:** Physiological indices, nitrogen (N), phosphorus (P), potassium (K), and chlorophyll (CHL) concentrations of *Pelargonium* leaves in plants inoculated (MICO+) or not (MICO_0_) with arbuscular mycorrhizal fungi (AMF) and grown for 28 (only electrolyte leakage) and 74 d in substrate amended with different rates of biochar (BC_0_, BC_30_, BC_70_), and fertilized with different rates of fertilizer (FERT_1_, FERT_2_).

Treatments	EL (%)		RWC (%)	CHLa	CHLb	CHLtot	N	P	K
					
				(g kg^-1^ DW)			(μg mg^-1^ DW)		
	
		Sampling time (DAT)								
	28		74						
**Biochar rate (BC)^1^**
BC_0_	0.9^b^	7.3	88^b^	0.32	0.13	0.46	12.3^b^	3.7^a^	1.8^c^
BC_30_	0.9^b^	7.2	89^ab^	0.34	0.14	0.49	14.4^b^	3.4^b^	2.6^b^
BC_70_	1.8^a^	6.7	90^a^	0.31	0.14	0.45	17.1^a^	1.9^c^	3.9^a^
**Fertilizer rate (F)^2^**
FERT_1_	1.0	6.4	89	0.31	0.13^b^	0.45^b^	12.2^b^	2.7^b^	2.6
FERT_2_	1.3	7.8	89	0.33	0.14^a^	0.49^a^	16.3^a^	3.4^a^	2.9
**AMF inoculation (I)**
MICO+	1.3	6.1^b^	90^a^	0.34	0.16^a^	0.49^a^	14.3^a^	3.2^a^	2.9^a^
MICO_0_	1.0	8.3^a^	88^b^	0.33	0.13^b^	0.45^b^	14.2^a^	2.8^b^	2.5^b^
**Significance^3^**
Biochar rate (BC)	*	NS	*	NS	NS	NS	**	**	*
Fertilizer rate	NS	NS	NS	NS	*	*	**	***	NS
AMF inoculation (I)	NS	**	*	NS	**	**	NS	*	*
BC*F	*	NS	NS	***	***	***	*	NS	NS
BC*I	NS	NS	NS	NS	NS	NS	NS	NS	NS
F*I	NS	NS	NS	NS	**	**	NS	NS	NS
BC*I*F	NS		NS	NS	NS	NS	NS	NS	NS	NS

^1^BC_*0*_, BC_*30*_, and BC_*70*_: 100:0, 70:30, and 30:70 (v:v) peat:biochar ratio, respectively.

^2^FERT_*1*_ and FERT_*2*_: fertilized with 140 and 210 mg L^-*1*^ of Nitrophoska^®^ Gold^®^, respectively.

^3^NS, *, **, and *** not significant or significant at *P* ≤ 0.05, *P* ≤ 0.01, and *P* ≤ 0.001respectively.

^a-b-c^Means in columns followed by the same letters are not significantly different according to the LSD test (*P* = 0.05). Means in columns without letters are not significantly different according to the LSD test (*P* = 0.05).

Biochar amendment and fertilizer rate significantly affected leaf nutrient concentrations (**Table 5**). Leaf N concentration was greatest in BC_70_ plants at both fertilizer rates and in BC_30_/FERT plants (**Figure 3C**). Compared with pure peat, increasing the proportion of biochar in the substrate decreased leaf P concentration by 8 and 50% and increased leaf K concentration by 44 and 116% in BC_30_ and BC_70_ plants, respectively. Increasing fertilizer rate increased leaf P but not K concentration (**Table 5**).

### Effect of AMF Inoculation on Plant Growth, Reproductive Traits, Nutritional Status, and Bio-Physiological Response

Inoculation with AMF significantly enhanced total plant leaf area (366 vs. 266 cm^2^), leaves per plant (19 vs. 16), flowers per plant (37 vs. 26), and flower number per cluster (8.4 vs. 6.2), and resulted in higher DW accumulation in leaves (2.1 vs. 1.7 g), stems (1.0 vs. 0.9 g), floral clusters (2.7 vs. 2.2 g), and on total a plant basis (6.8 vs. 5.7 g; **Table 4**). At 74 DAT, MICO+ plants had a lower EL physiological stress index and higher RWC index compared with MICO_0_ plants (**Table 5**). AMF inoculation increased leaf P and K concentrations (**Table 5**) and, in media fertilized at the highest rate, increased the total CHL and CHL b concentrations of leaves (data not shown, treatment significance in **Table 5**).

### Effect of Biochar, Fertilizer Rate, and AMF Inoculation on Leaf and Flower Color

All the measured leaf colorimetric parameters (lightness, chroma, and hue angle) were significantly affected by the fertilizer rate, with significant interactions between fertilizer rate and BC treatment for L* and C* index (**Table 6**). As expected, increasing fertilizer rate increased h°, which corresponds to a greater intensity of greenness and a desirable decrease in yellowness. Leaf L* and C* values were higher with the lowest fertilizer rate (**Table 6**), especially in BC_0_ (38 and 22, respectively) and BC_30_ (37 and 21, respectively) media, indicating leaves were a lighter green color and had a tendency to more red pigmentation. AMF inoculation decreased the L* and C* indexes. Flower color parameters were affected by BC treatment. The h° index, whose higher values are related to higher red color intensity of petals, was reduced in 70% biochar-added media. The vivid red color and brightness of flowers (low L and C indices values) were improved (**Table 6**) by the highest fertilizer rate.

**Table 6:** Color parameters of leaves and flowers of *Pelargonium* plants inoculated (MICO+) or not (MICO_0_) with AMF and grown for 74 d in substrate amended with different rates of biochar (BC_0_, BC_30_, BC_70_), and fertilized with different rates of fertilizer (FERT_1_, FERT_2_).

Treatments	Leaves			Flowers		
	L*	h*	C*	L*	h*	C*
**Biochar rate (B)^1^**
BC_0_	39.8	111.6	25.5	30.2	31.1^ab^	60.0
BC_30_	38.9	112.2	24.4	30.8	32.0_a_	60.8
BC_70_	38.3	113.8	22.7	30.7	29.9_b_	57.7
**Fertilizer rate (F)^2^**
FERT_1_	39.9_a_	111.3_b_	25.5_a_	30.9_a_	31.6	60.8_a_
FERT_2_	38.2_b_	113.7_a_	23.0_b_	30.3_b_	30.6	58.6_b_
**AMF inoculation (I)**
MICO+	38.2_b_	112.9	23.2_b_	30.7	30.2	59.4
MICO_0_	39.8_a_	112.1	25.3_a_	30.4	30.9	59.8
**Significance^3^**
Biochar rate	NS	NS	NS	NS	*	NS
Fertilizer rate	***	***	***	*	NS	*
AMF inoculation	**	NS	**	NS	NS	NS
B*I	NS	NS	NS	NS	NS	NS
B*F	***	NS	***	NS	NS	NS
I*F	NS	NS	NS	NS	NS	NS
B*I*F	NS	NS	NS	NS	NS	NS

^1^BC_*0*_, BC_*30*_, and BC_*70*_: 100:0, 70:30, and 30:70 (v:v) peat:biochar ratio, respectively.

^2^FERT_*1*_ and FERT_*2*_: fertilized with 140 and 210 mg L^-1^ of Nitrophoska^®^ Gold^®^, respectively.

^3^NS, *, **, and *** not significant or significant at *P* ≤ 0.05, *P* ≤ 0.01, and *P* ≤ 0.001, respectively.

^a-b^Means in columns followed by the same letters are not significantly different according to the LSD test (*P* = 0.05). Means in columns without letters are not significantly different according to the LSD test (*P* = 0.05).

## Discussion

In this study the replacement of peat with 30% biochar does not modify *Pelargonium* growth compared with pure peat. By increasing the fertilization rate, the vegetative growth and quality of plants was improved in both BC_30_ media and pure peat, with plants being taller, with more branches and leaves (**Figure 1**), as well as having a greater dry mass (**Figure 2**), and more intense and vivid color of leaves and petals (**Table 6**). In particular BC_30_, when in combination with FERT_2_ treatment, was more effective in enhancing the *Pelargonium* leaf number and flowering performance (**Figures 1D–F**). The highest N and CHL concentrations observed in leaves from the BC_30_ plants at the highest fertilizer rate (**Figures 3B,C**) underlines a better plant nutritional status that could be considered the reason for the promotion of leaf formation and flowering.

In contrast, with the highest fertilizer rate the BC_70_ substrate depressed plant growth and development compared with BC_0_ and BC_30_ substrates (**Figures 1** and **2**).

The positive results obtained with the low biochar rate (30%) under higher fertilization regime, are in agreement with those obtained by Graber et al. (2010) who found an increase in growth and productivity (flower number) of well-fertilized bell pepper by the addition of biochar to a coconut fiber:tuff mix. They hypothesized that their positive results were related to the development of a more beneficial microbial community and/or to biochar-induced systemic resistance to diseases. Similar results were obtained with rice hull gasified biochar at 10% mixture with peat (v:v), which increased shoot dry mass of *Pelargonium* plants as well as availability of P and K (Altland and Locke, 2013). In another experiment, *Calathea rotundifolia* plants showed improved leaf and total biomass and leaf area when grown in 50% green waste pyrolyzed biochar added peat medium (Tian et al., 2012) compared to 100% peat. Other works report only improved plant height in medium amended with wood pyrolyzed biochar (1–5%, w:w) for tomato (Graber et al., 2010) and zinnia (10–30% v:v; Kadota and Niimi, 2004), and with gasified biochar (25% v:v) for tomato and marigold (5–15% v:v; Vaughn et al., 2013). The observed changes in the mixtures compared to pure peat for BD, AS, and water-filled porosity (WFC) do not seem to be directly responsible for the differences in plant responses. Indeed, at both rates of biochar the BD was kept within the acceptable range (BD less than 0.40 g cm^-3^), while TP remained close to the lower limit (TP ≈ 85%; **Table 2**), as proposed by de Boodt and Verdonck (1972). The greater amount of the largest particles (>2 mm; **Table 1**) probably enhanced the mixtures AS compared to pure peat by bringing the values of mixtures close to the optimal range of 20–30%. However, it also reduced the WFC and water content in the BC_70_ substrate at the end of the trial (**Table 2**). Despite these results, the BC_70_ plants had well hydrated tissues (high RWC, **Table 5**) leading us to hypothesize that water availability was not responsible for the growth depressive effect caused by BC_70_ treatment.

The EC of the media rose with increasing biochar amendment rate, however, it remained, on average, close to the optimal range for *Pelargonium* cultivation (**Table 2**). Nevertheless, EC have increased to over the optimal range in the 70% biochar treatment at the higher fertilizer rate. In fact, reduced shoot growth in BC_70_ plants compared to BC_30_ and BC_0_ plants 28 DAT (**Figures 2A,B**) could be related to a rise in EC causing osmotic stress in BC_70_ plants. The higher EL values at 28 DAT in the BC_70_/FERT_2_ treatment (**Figure 3A**) supports the theory that plants may have been under greater osmotic stress with a consequent reduction in growth rate. The subsequent depletion of solutes that occurred later in the growing cycle due to plant uptake and leaching may have overcome this effect by 74 DAT.

In BC_0_ pH was slight higher than the optimal values for *Pelargonium* (6.4–6.5 pH, Andrews and Hammer, 2006). Irrespective of the rate, the addition of biochar resulted in a quite substantial pH increase (≈ +1 pH unit) compared to pure peat, thus resulting in an increased difference from the optimal values. However, no pH differences were observed between the 30 and 70% biochar substrates, therefore the different plant responses observed in these two substrates cannot be considered linked to substrate pH.

In all treatments the final leaf N concentration was below the level recommended as optimal for *Pelargonium* cultivation (30–40 g kg^-1^ DW, Krug et al., 2006), so we can hypothesize that plant were in a N deficiency status. This result was not surprising given the low level of fertilizer applied in this study. In order to test the difference in nutrient availability between pure peat and biochar amended substrates we deliberately performed fertilization at low rates and only at the beginning of the experiment. The less severe N deficiency observed in BC_30_/FERT_2_ and BC_70_ plants (**Figure 3B**) might have occurred as a consequence of an improved retention capacity of nutrients in the biochar mixtures, as also reported by other authors (Asai et al., 2009; Zhang et al., 2014). However this result, in agreement with Cao and Harris (2010) and Altland and Locke (2013), indicates a low biochar N availability. The low N availability can be linked to (i) the low biochar N concentration (2.9 g kg^-1^), due to the high temperature of gasification (Laird et al., 2011; Peterson and Jackson, 2014), and to (ii) the microbial biomass immobilization, considering the high C/N ratio of the used biochar (Rajkovich et al., 2012).

In BC_70_ plants also the lower microbial activity and biomass, as a consequence the high levels of the biocidal compounds in the biochar produced during gasification, might have resulted in a lower immobilization of N. Although not constituting direct evidence, the observed low frequency of mycorrhizal colonization in the BC_70_ treatment (**Table 3**) indicates the inhibitory effect of this substrate to microorganisms. The reduction of mycorrhizal symbiosis only with the highest biochar rate is in agreement with Warnock et al. (2010), who report a decrease in AMF root colonization above a certain threshold of biochar application.

Leaf concentration of K and P were considered adequate for mature zonal geranium leaves (25–30 and 1.7–3.5 g kg^-1^ DW, respectively, Krug et al., 2006), however, compared to pure peat, leaf P was slightly reduced in BC_30_, and it dropped close to sub-optimal values in BC_70_ (**Table 5**).

The high pH values did not favor the P availability in either BC_30_ or BC_70_ mixtures, thus the higher leaf P concentration in BC_30_ compared with BC_70_ plants might be related to the higher mycorrhizal colonization which improved plant P acquisition (**Table 3**). Hammer et al. (2014) reported that AM fungal hyphae access microsites within biochar, that are too small for most plant roots to enter, and may mediate plant P uptake from the biochar. Moreover it is well-known that mycorrhizal symbiosis improves efficiency in P acquisition by plants, often resulting in growth promotion (Smith and Read, 2008).

It is known that N and P are involved in the regulation of several photosynthetic processes and that N:P ratio very important for plant growth (Güsewell, 2004). Nitrogen and/or P deficiency induces the reduction in photosynthetic capacity which can mostly be attributed to the dysfunction of the Calvin cycle and limitations in nutrient supply to the chloroplasts (Lima et al., 1999). The higher N:P ratio (8.9) in BC_70_ plants due to P deficiency, compared with BC_0_ and BC_30_ plants (3.7 on average) could be the explanation of the observed difference in growth between them.

Although root infection by indigenous AM fungi occurred in all cases, the frequency of root colonization was higher (**Table 3**) and higher effectiveness in improving *Pelargonium* production (**Table 4**) was achieved by inoculation with selected AMF, confirming reports by other authors on potted ornamental plants (Navarro et al., 2011, 2012; Asrar et al., 2012).

In agreement with a number of reports (see Smith and Read, 2008, for a review) our results point out how AMF inoculation results in plants with an improved nutritional status (higher leaf P and K concentration), unstressed leaf tissues (lower EL and higher RWC values; **Table 5**) and with an improved growth (higher dry biomass, greater floral clusters, larger, more abundant leaves; **Table 4**), greener leaves (high CHL content, low L* and C*) and more intensively colored flowers (**Tables 5 and 6**).

## Conclusion

This research demonstrates that biochar obtained from gasified wood feedstock, although causing adversely high pH conditions in the peat mixtures, can be applied in nursery/potted plant production provided that the proportion in the peat mixture does not exceed 30%. In the media amended with 30% rate of biochar the mycorrhizal root colonization seems to improve P acquisition under non-optimal pH conditions. The available N from biochar is not adequate to support optimal potted *Pelargonium* growth. N fertilization in 30% amended medium should be further studied in research to assess the optimal N rate, also considering the possible rise in EC. At a 70% rate biochar could have negatively affected plant growth through osmotic stress and/or the inhibition of mycorrhizal activity, beneficial for P nutrition. The inoculation of media with selected AMF did not interact with the biochar proportion or with fertilization level, contributing to the best performance in the 30% biochar amended medium with the highest fertilizer rate.

Additional research will be needed to better elucidate the relationship between biochar proportion, fertilization rate and the chemical characteristics of biochar-peat mixtures, as well as the role of mycorrhizal symbiosis in the mineral nutrition of plants grown in biochar mixtures.

## Conflict of Interest Statement

The authors declare that the research was conducted in the absence of any commercial or financial relationships that could be construed as a potential conflict of interest.
